# Successful Pregnancy Outcome of a Suspected Molar Pregnancy After a Trial of In Vitro Fertilization/Intracytoplasmic Sperm Injection (IVF/ICSI): A Case Report and Literature Review

**DOI:** 10.7759/cureus.81693

**Published:** 2025-04-04

**Authors:** Ghufran S Alsaffar, Zainab H Madan, Mahmoud A Alsalama, Mohamed Taman

**Affiliations:** 1 Department of Obstetrics and Gynecology, Mansoura University Hospitals, Mansoura, EGY

**Keywords:** beta-hcg, case report, hydatidiform mole, icsi, ivf, molar pregnancy, ultrasound

## Abstract

Twin pregnancy with a complete hydatidiform mole and a co-existing fetus (CHMCF) is a rare situation and may occur after using assisted reproductive technologies (ARTs). These pregnancies are associated with severe maternal and fetal complications, and their management is challenging. Ultrasound (US) and magnetic resonance imaging (MRI) are commonly used tools for the diagnosis of suspected molar pregnancy. However, they are not 100% sensitive or specific, and pregnancy could end with a healthy baby. Histopathological examination is considered the gold standard for confirming the diagnosis of hydatidiform mole. Here, we describe a case with CHMCF after an intracytoplasmic sperm injection (ICSI) trial. A 36-year-old primigravida underwent two embryo transfers after an ICSI trial after a period of 14 years of primary subfertility. She was suspected of having one healthy intrauterine pregnancy and co-existing suspected molar pregnancy by US and beta-human chorionic gonadotropin (beta-hCG) level. After counseling, she decided to continue the pregnancy, and then she developed rising blood pressure and succeeded in delivering a healthy baby in the 37th week of gestation by elective cesarean section (CS). This case and literature review highlight that CHMCF may still occur even if the pregnancy was achieved by ICSI, and under close follow-up, successful outcomes could be achieved in such pregnancies.

## Introduction

Hydatidiform mole is a gestational trophoblastic disease with two different genetic forms: complete and partial hydatidiform mole. It happens mainly because of having chromosomally malformed oocytes or sperms, which are not commonly tested during the in vitro fertilization/intracytoplasmic sperm injection (IVF/ICSI) procedure. A twin pregnancy with a complete hydatidiform mole and a co-existing fetus (CHMCF) is a rare situation, with an estimated frequency of 1 in 22,000-100,000 pregnancies. CHMCF can also occur after IVF and ICSI [1].

The incidence of twin pregnancies with a CHMCF following IVF and embryo transfer has been reported to be not higher than that of the general population. Pregnancy is usually terminated, but the continuation of pregnancy could be done, particularly after subfertility treatment [2].

Compared to partial hydatidiform moles, in diagnosing complete hydatidiform moles ultrasound (US) examinations are more reliable. Detecting molar pregnancy with ultrasonography remains a diagnostic challenge, particularly for patients with partial hydatidiform moles [3]. Newhouse et al. (2022) reported that the specificity of ultrasonography for diagnosing hydatidiform moles was 92.6%, and the sensitivity was 52.2% [3]. A pelvic magnetic resonance imaging (MRI) could be requested to detect any myometrial invasion and to confirm US results. It has two main purposes: it describes the extent of the mole's invasion and searches for distant metastases [4]. Histopathological examination is considered the gold standard for confirming the diagnosis of hydatidiform mole [3].

## Case presentation

A 36-year-old primigravida underwent a trial of ICSI following the failure of the previous four trials of IVF after a period of 14 years of primary subfertility and transferred two fresh blastocysts on day five. During her routine antenatal care at 12 weeks of gestation, some changes were noticed by the US at the site of the placenta, and she was referred to our tertiary health care center for multidisciplinary team evaluation and a decision either for the continuation or the termination of the pregnancy.

**Figure 1:**
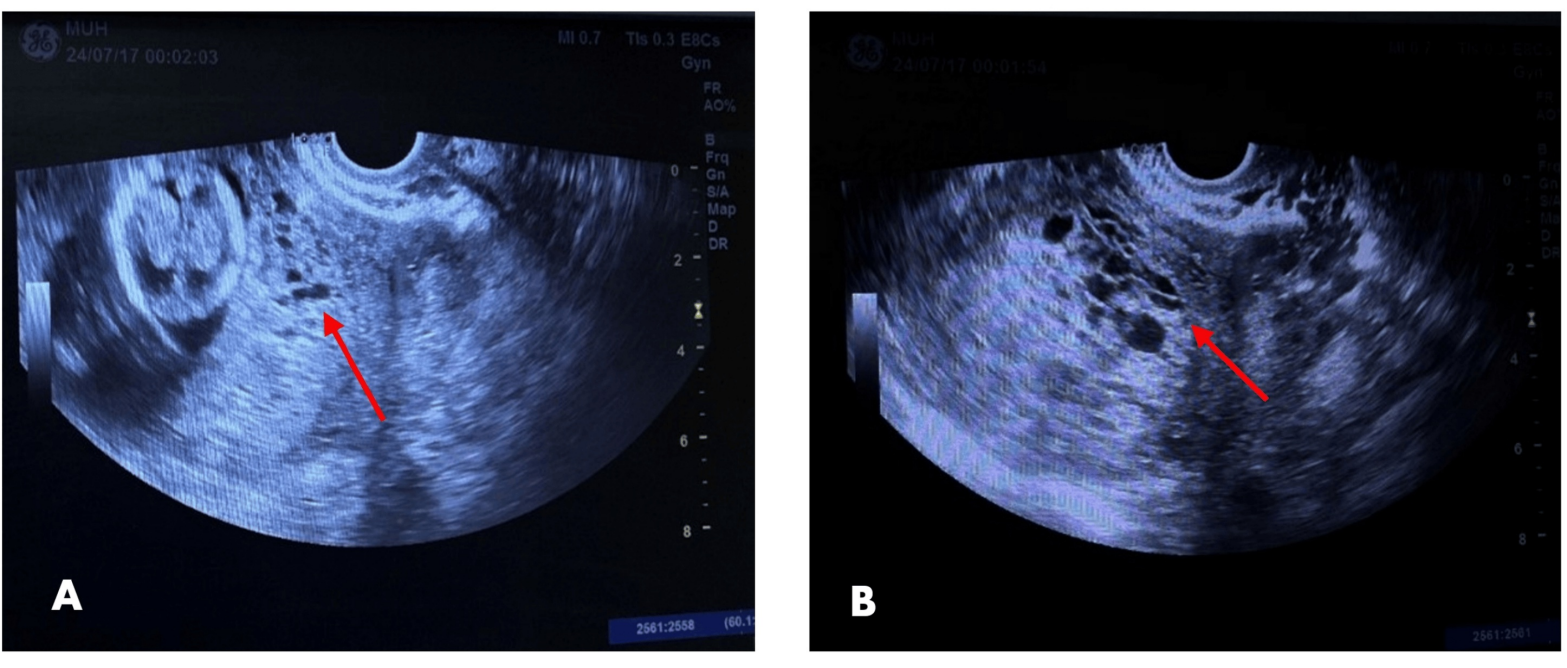
Ultrasound scan at 12 weeks of gestation (A) Ultrasound image showing a grape-like appearance of a coexisting fetus (suspected to be a hydatidiform mole) located at the uterine fundus (arrow). (B) Sonographic image showing placenta with multiple cystic spaces (arrow).

**Figure 2:**
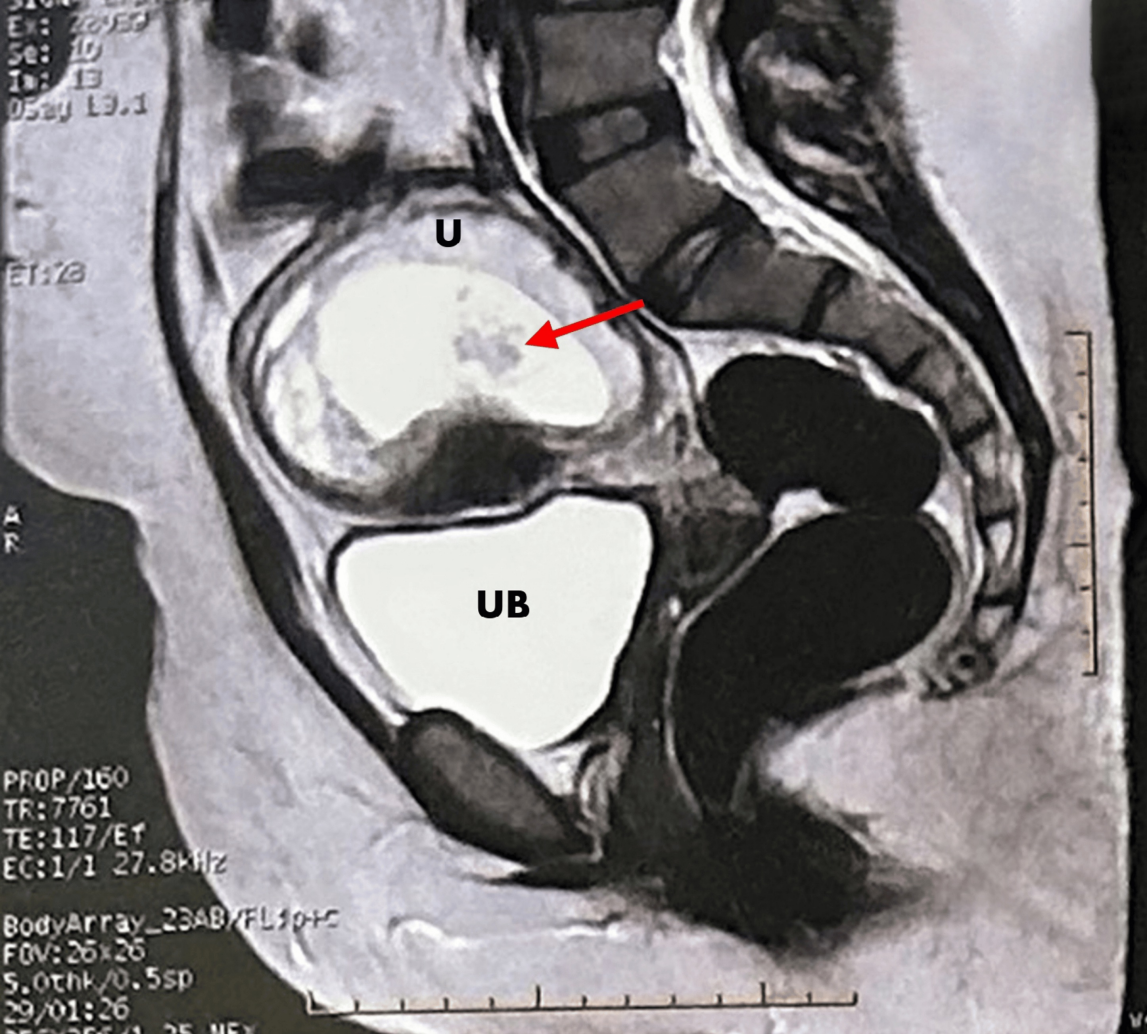
T2-weighted MRI scan T2-weighted MRI scan showing a completely adherent molar-like lesion (multi-cystic appearance) encasing the fetal sac with foci of intra-lesional blood signal inside measuring about 4.8*3*5.8 cm, thin rim of subchorionic hematoma 3 mm with no extrauterine extension and no pathological pelvic lymph nodes. MRI: magnetic resonance imaging, UB: urinary bladder, U: uterus

The US demonstrated a grape-like appearance of a coexisting fetus located at the uterine fundus, raising a high suspicion of a hydatidiform mole (Figure 1). Serum beta-human chorionic gonadotropin hormone (beta-hCG) was 117,300 mIU/ml (reference range: 1,400-53,000 mlU/ml). A non-contrast MRI of the pelvis with different sequences was done, showing a completely adherent molar-like lesion (multi-cystic appearance) encasing the fetal sac with foci of intra-lesional blood signal inside measuring about 4.8*3*5.8 cm, thin rim of subchorionic hematoma 3 mm with no extrauterine extension and no pathological pelvic lymph nodes (Figure 2).

After reevaluation by a multidisciplinary team and counseling of the patient, a decision to continue the pregnancy was made.

**Figure 3:**
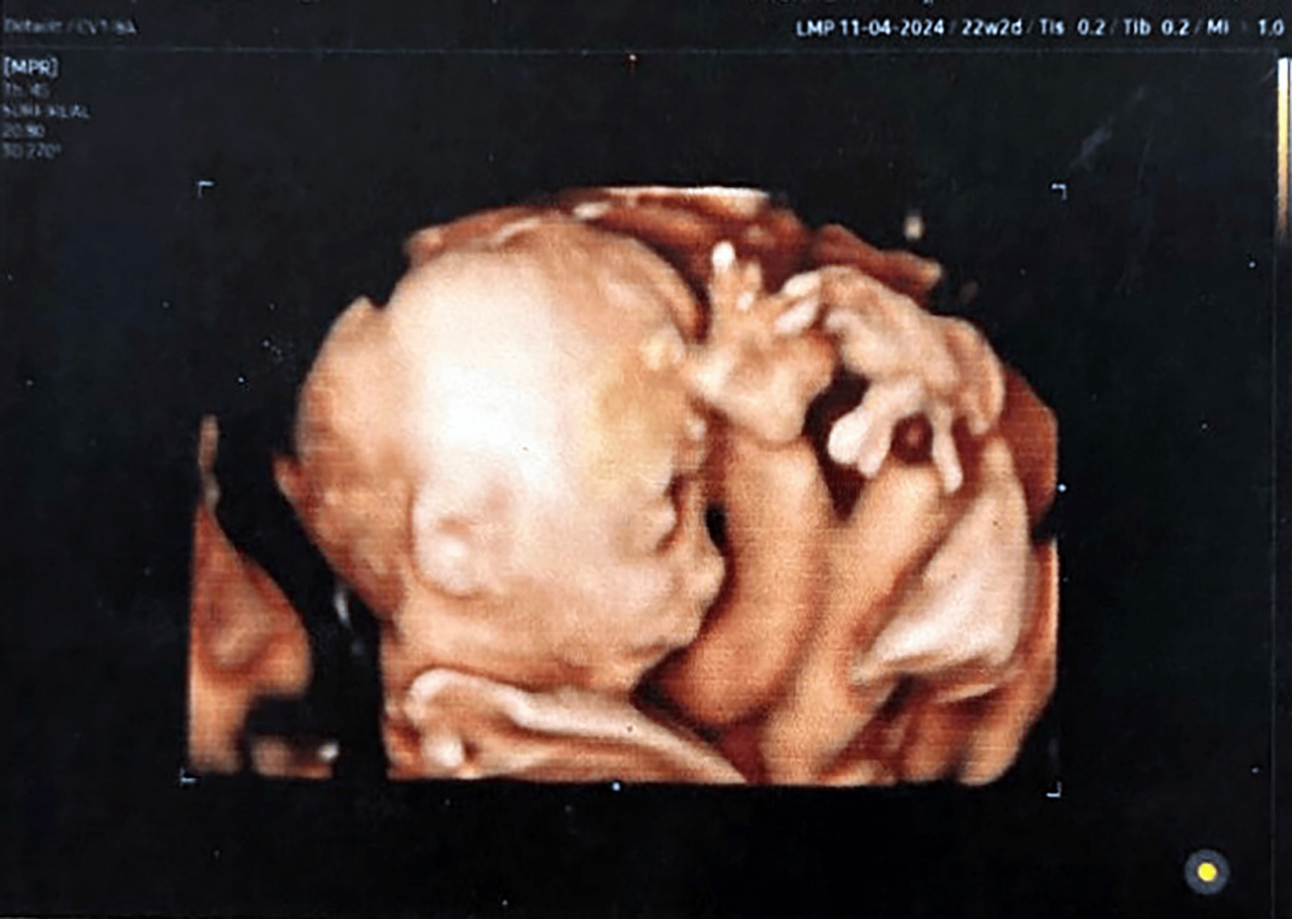
3D/4D Ultrasound scan at 22 weeks of gestation showing no structural anomalies.

During her routine antenatal care, the patient underwent an anatomical scan at 21 weeks of gestation, showing a single viable healthy baby with a fetal size appropriate for gestational age (GA). The placenta was posterior, the liquor was adequate, and the sonolucent spaces were still persistent inside the placenta. A further evaluation using 3D/4D ultrasound was done at 22 weeks of gestation with no detected ultrasonographic markers suspecting any fetal congenital anomalies (Figure 3).

At 32 weeks of gestation, the patient started to develop symptoms of headache and blurring of vision, and her blood pressure was estimated to be 170/100 mmHg. She was started on labetalol 200 mg twice daily and received magnesium sulfate 4-6 g as a loading dose slowly intravenously (IV) over 20 minutes, followed by 1 g per hour maintenance therapy for 24 hours for fetal neuroprotection. She continued her follow-up until 37 weeks when an elective cesarean section (CS) was performed. She delivered one living female baby, weighing 3200 g, evaluated by a neonatologist with an APGAR score of 9 out of 10 at 5 minutes. The baby's clinical examination and initial assessment by the neonatologist were normal.

After delivery, a large placenta measuring 20*14*7 cm was sent for histopathological examination. Microscopic examination revealed multiple chorionic villi covered by inner cyto and outer syncytiotrophoblastic cells with areas of interstitial hemorrhage. Slides prepared from the umbilical cord and fetal membranes were free with no detected significant chorioamnionitis and no detected atypia or malignancy in the examined material. Monitoring beta-hCG revealed a decline in the levels of the hormone after delivery, with no myometrial invasion on the follow-up ultrasound, as well as beta-hCG normalization after 3 months of follow-up.

## Discussion

The coincidence of a hydatidiform mole with a viable fetus represents one of the rarest conditions after ICSI cycles. After reviewing the literature in addition to the present case, we include nine cases of complete molar pregnancy and two cases of partial molar pregnancy after IVF (Table 1) [1,5-11].

**Table 1:** The reported cases of hydatidiform mole and co-existing live fetus after ICSI in the literature. ICSI: intracytoplasmic sperm injection, CHM: complete hydatidiform mole, PHM: partial hydatidiform mole, NM: not mentioned, CS: cesarean section, VD: vaginal delivery, TAH: total abdominal hysterectomy, IUFD: in utero fetal death, UTI: urinary tract infection.

Authors	Type of mole	Maternal Age	Gestational age at Diagnosis (Weeks)	Gestational age at Delivery (Weeks), pregnancy outcome	Birth weight, Newborn outcome	Complications during pregnancy	Histopathology	Persistent trophoblastic disease
V. Alpay et al. [1]	CHM	33	12	26, CS	625 g, alive	Preeclampsia, Preterm delivery	Consistent with CHM	Yes (lung metastasis)
Kashani E et al. [5]	CHM	29	19	19, termination	Dead	Preeclampsia, IUFD	Consistent with CHM	Yes (no metastasis)
Haruka Hamanoue et al. [6]	CHM	40	16	33, CS & TAH	1544 g, alive	Vaginal bleeding, Preterm delivery	Consistent with CHM	No
Ashraf Moini et al. [7]	CHM	39	15	39, CS	3150 g, alive	No	Consistent with CHM	No
Bruchim et al. [8]	CHM	28	7	41, VD	3240 g, alive	Gestational hypertension	Consistent with CHM	No
	CHM	25	NM	26, CS	875 g, alive	Vaginal bleeding, Preterm labor	Consistent with CHM	No
Kenan Dolapcioglu et al. [9]	CHM	34	13	29, CS	1180 g, alive	Vaginal bleeding, Preterm delivery	Consistent with CHM	No
Asha R. Rao et al. [10]	PHM	24	13	32, CS	1500 g, alive	Gestational hypertension, Preterm delivery	Consistent with PHM	No
	PHM	27	12	27, spontaneous expulsion	Dead	IUFD	-	No
	CHM	29	12	31, CS	1670 g, alive	Hydronephrosis, UTI, Preterm delivery	Consistent with CHM	No
Tiago José Ferraz et al. [11]	CHM	39	12	14, termination	-	Hyperthyroidism	Consistent with CHM	Yes (no metastasis)
Present case	CHM	36	12	37, CS	3200 g, alive	Gestational hypertension	Inconsistent with CHM	No

The mean age of reported cases was 31 years, and four of them were pregnant above the age of 35 years [6,7,11]. Our patient was 36 years old. Complete molar pregnancy was reported to happen in extremes of reproductive ages (18-40 years) [12]. Three cases were reported in the first pregnancy [5,9,10], and eight cases were multigravida [1,6-8,10,11]. CHMCF was suspected in the first pregnancy of our patient. Nahid Mirza et al. (2022) reported that among the participants of their study who suffered from molar pregnancy, 65% were multipara and 35% were primigravida [13]. Anyanwu M et al. (2020) in Gambia from 2016-2018 also reported multiparous women predominance [14]. Nine cases were twin pregnancies with CHMCF [1,5-11] and two were twin pregnancies with partial hydatidiform mole and co-existing fetus (PHMCF) [10]. Twin pregnancies with a hydatidiform mole and a healthy fetus give rise to complex clinical considerations, particularly when the pregnancy is highly desired.

Maternal complications and the necessity of termination of pregnancy are important matters in clinical management. Five cases of CHMCF and one case of PHMCF were complicated by pre-term labor [1,6,8-10]. Liang H et al. (2022) reported that the incidence of preterm delivery (33, 33%) for PHMCF was lower than that of CHMCF (54, 55) [15]. Our patient was delivered at 37 weeks. Moreover, two cases were complicated with early-onset preeclampsia, and subsequently, the pregnancy was terminated at 26 weeks of gestation due to preterm labor [1] and 19 weeks of gestation due to intrauterine fetal death [5,16]. Three cases were complicated with vaginal bleeding [6,8,9]. Although vaginal bleeding is the most common complication in molar pregnancies, it seems to be less common in molar pregnancies associated with a co-existent fetus [1,5,11].

The most challenging aspect of counseling these couples is the possible risk for persistent gestational trophoblastic disease (pGTD), which ranges from 19.0% to 62.5% [17]. However, pregnancy termination does not reduce the chance of developing gestational trophoblastic disease following CHMCF [18], therefore, the diagnosis of pGTD was found in three cases [1,5,11], and two cases received single-agent chemotherapy with methotrexate. In the follow-up, serum beta-hCG levels returned to normal values, and complete remission was achieved [1,5].

Most of the patients had elevated beta-hCG levels, and all exhibited ultra-sonographic features consistent with molar pregnancy. In cases of hydatidiform mole, beta-hCG levels are typically elevated beyond the normal pregnancy range. For a complete mole, beta-hCG levels often exceed 100,000 mIU/mL. In contrast, a partial mole may present with beta-hCG levels within the range associated with normal pregnancy, making clinical diagnosis more challenging [19]. US examination is typically a reliable method of diagnosing hydatidiform mole and a co-existing fetus (HMCF). However, this condition is often missed at early gestational age because abnormal placental echoes are often mistaken as subchorionic hematoma, placental hemangioma, and accidental hemorrhage, which could be differentiated easily using 2D US, associated with Doppler examination and MRI. Liang H et al. (2022) reported that only 47.06% (8/17) of patients had been diagnosed before 12 weeks of gestation. Therefore, more investigation should be done to follow the development of a subchorionic hematoma when it is diagnosed in the first trimester. HMCF may be mistaken for placental mesenchymal dysplasia (PMD), which is a rare placental vascular anomaly because they have similar US characteristics. In this study, five cases of pathological findings were inconsistent with HMCF and instead met the criteria of PMD [15].

MRI plays a role in the detection and evaluation of hydatidiform moles. It also gives thorough tissue characterization, which is useful in determining the nature of molar tissues. On T2-weighted images, a complete mole shows as a heterogeneous mass with high signal intensity, often containing multiple cystic spaces. This imaging detail helps in accurate diagnosis and management planning [4].

Histopathology was done in all cases, and it was consistent with hydatidiform mole (diffuse mature, edematous, cystic chorionic villi), although in our present study histopathology revealed placental tissue with no significant pathological changes (multiple chorionic villi covered by inner cyto and outer syncytiotrophoblastic cells with areas of interstitial hemorrhage). Histologically, an enlarged villous trophoblast with cystic "swollen" villi is a characteristic of both complete and partial hydatidiform moles. Complete moles have no embryonic or fetal tissue at all, whereas partial moles have [20].

It is difficult to diagnose a molar placenta during an ongoing pregnancy. This case highlighted two primary features: first, US alone may not be sufficient for accurate management, despite being a good diagnostic technique in molar pregnancies; and second, complete molar pregnancies may still occur, even if the pregnancy was achieved by ICSI. The present case shows a favorable pregnancy outcome for both the mother and the fetus. As highlighted in the literature review, such pregnancies are associated with serious complications, and patients should be informed about the risks before deciding to terminate or continue the pregnancy. In parallel, we believe that these pregnancies should be continued and closely monitored unless there are fetal anomalies or death, as well as maternal complications.

## Conclusions

Pregnancies following IVF could be associated with some placental hydropic changes, particularly in cases of multiple pregnancies. US could be used as a first tool for diagnosis of suspected molar pregnancy, and MRI could be used for more confirmation; however, it’s not 100% sensitive or specific for diagnosis and it could end with a healthy baby and healthy placenta. Counseling of the patient about the continuation or termination of pregnancy should be guided by strong evidence of having associated congenital malformations. However, multicentric studies should be conducted to test the specificity and sensitivity of different diagnostic tools in diagnosing suspected molar pregnancy to be used as guidelines for deciding the continuation or termination of pregnancy.
